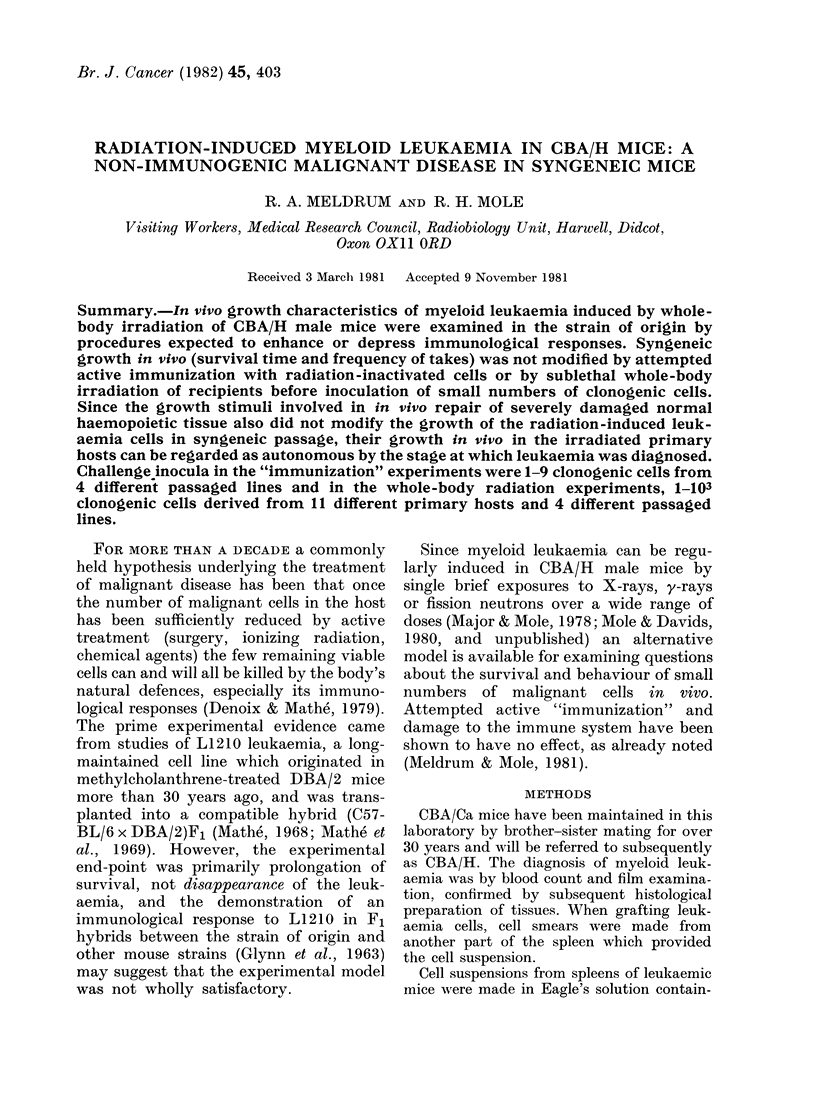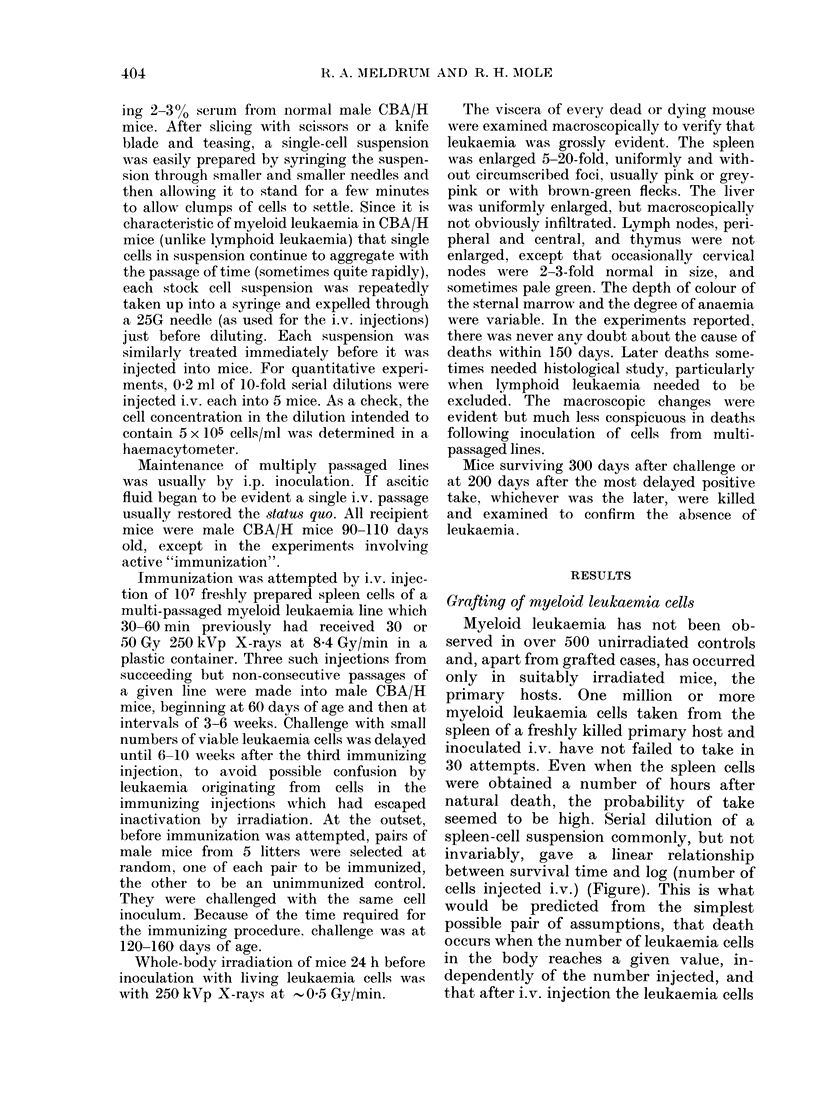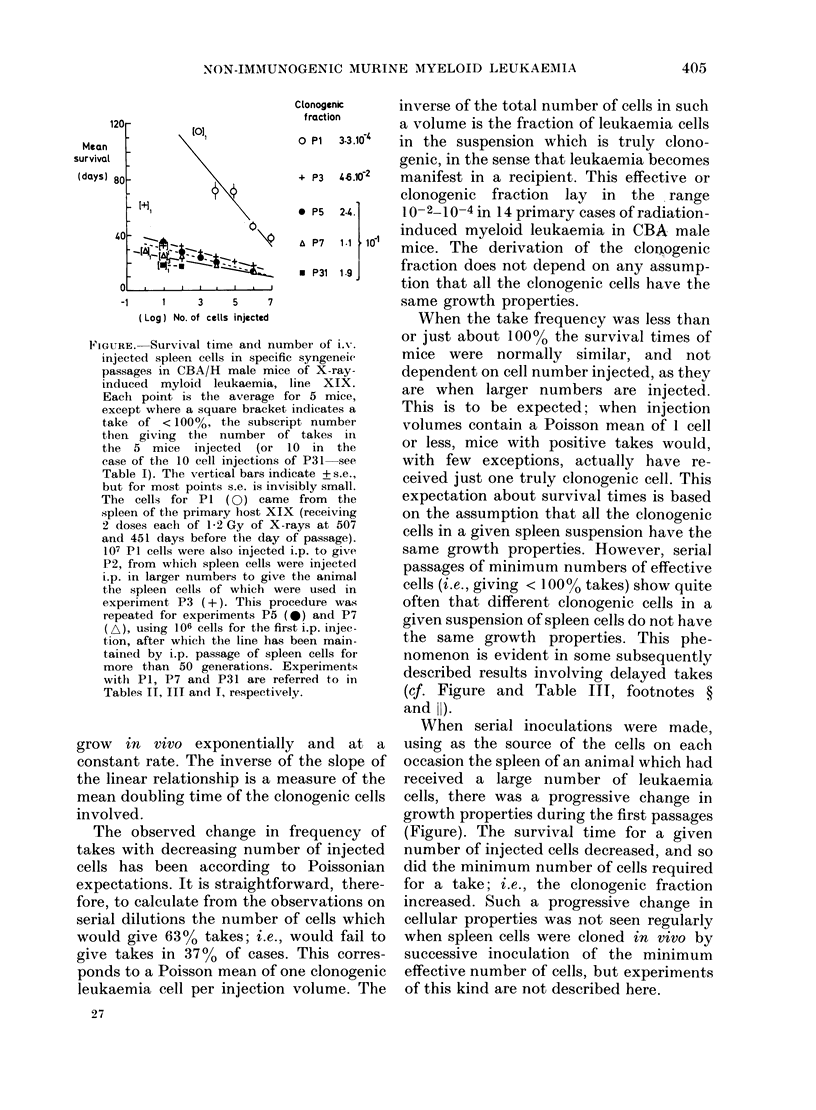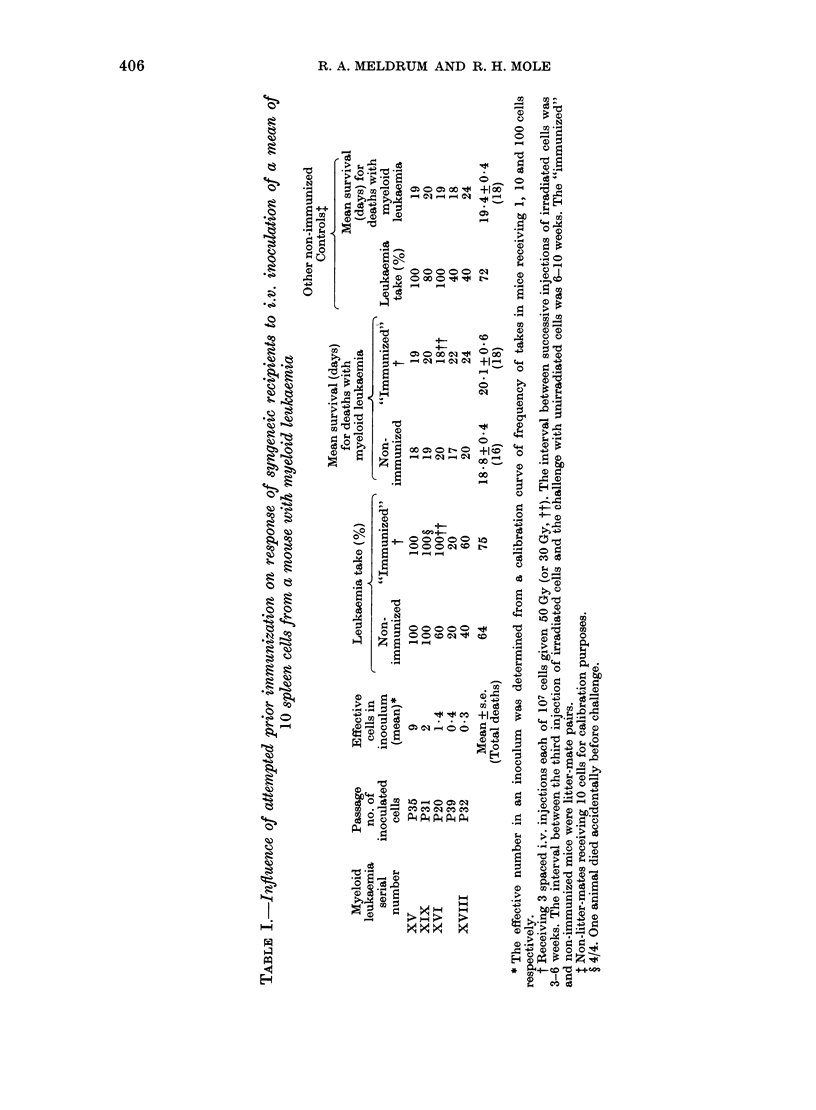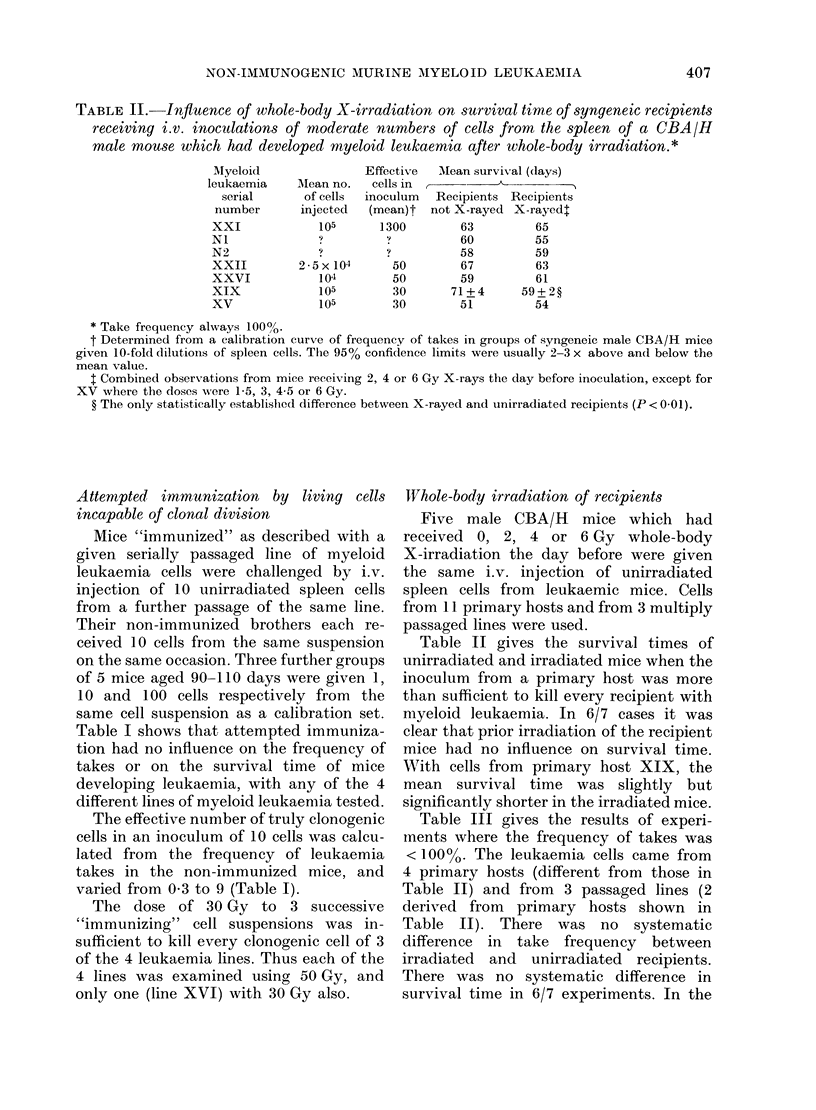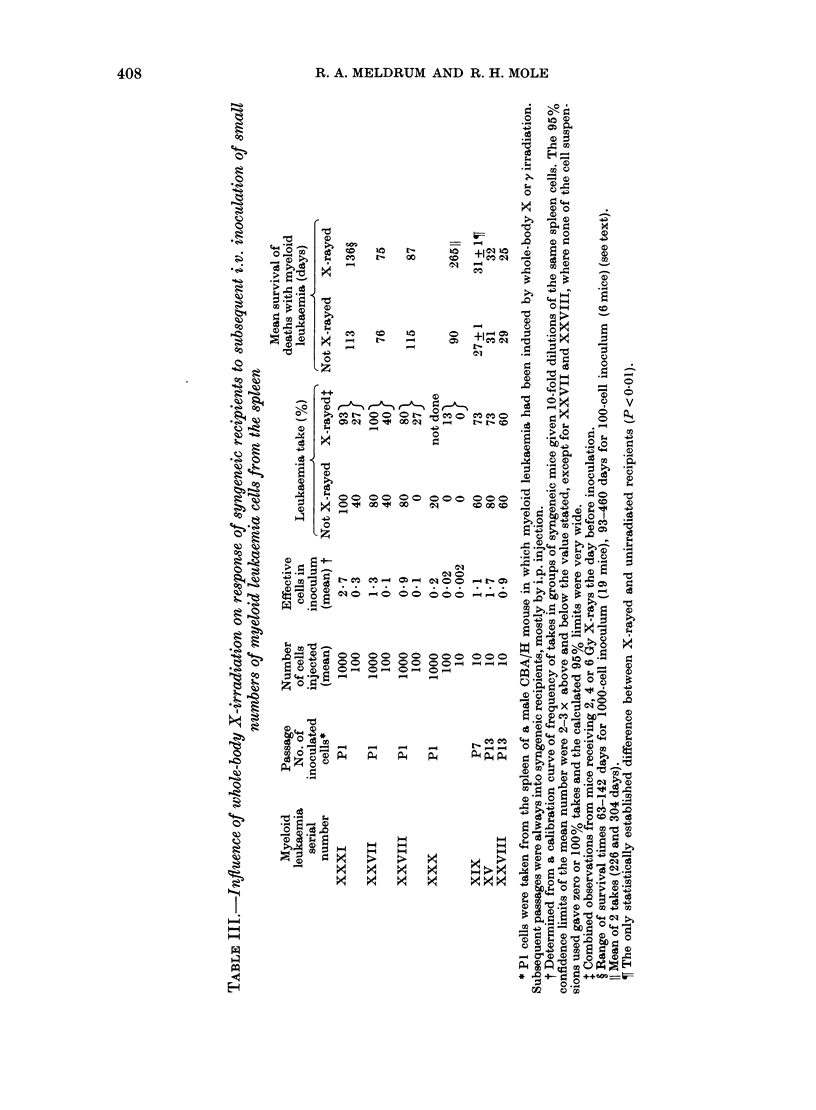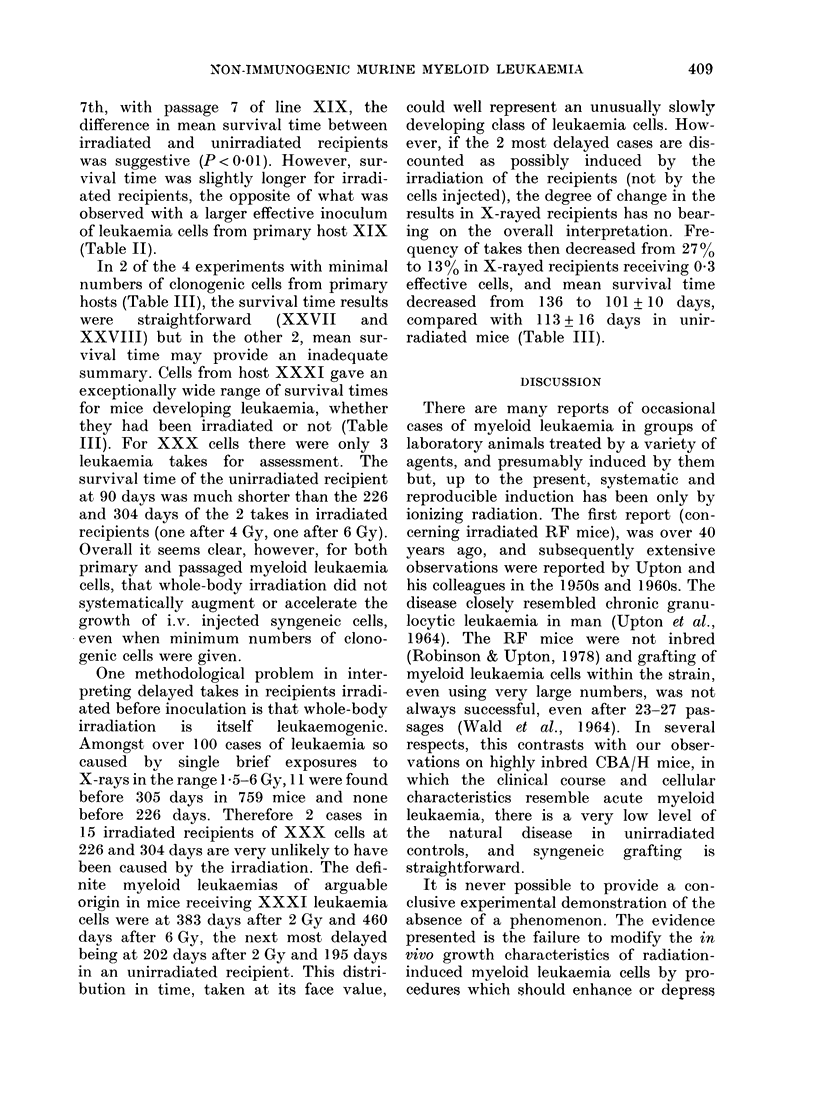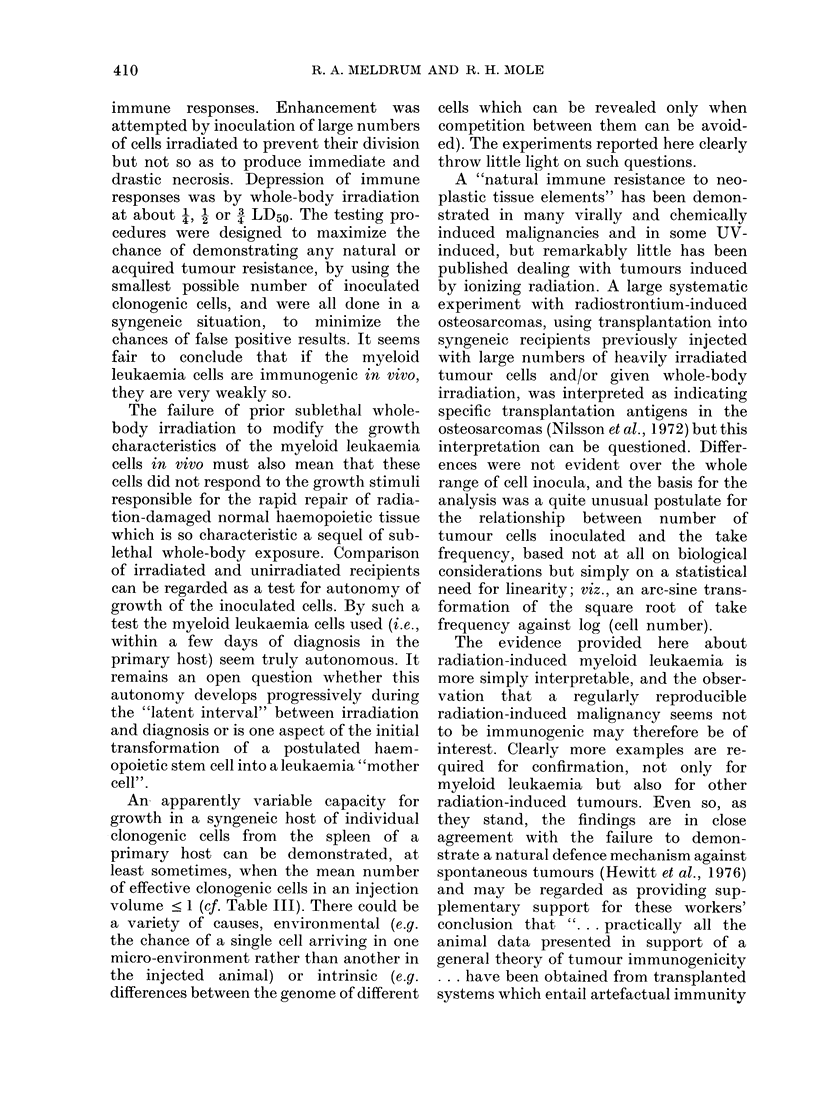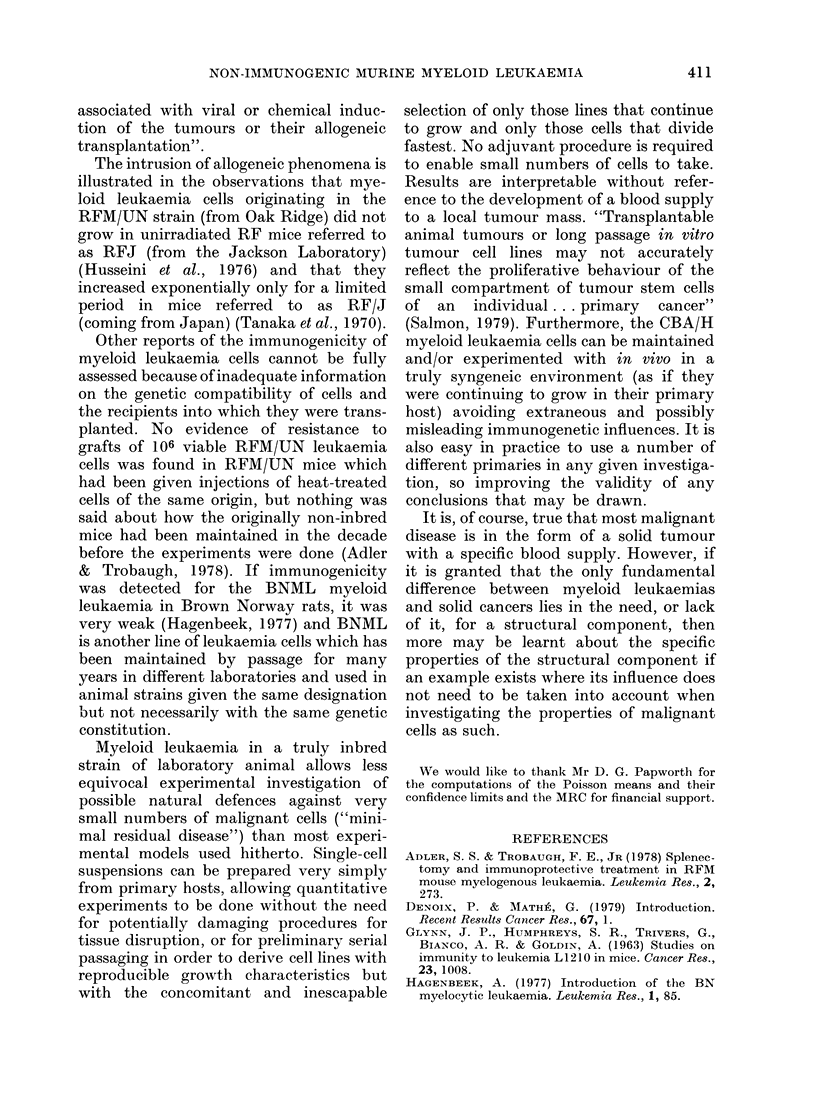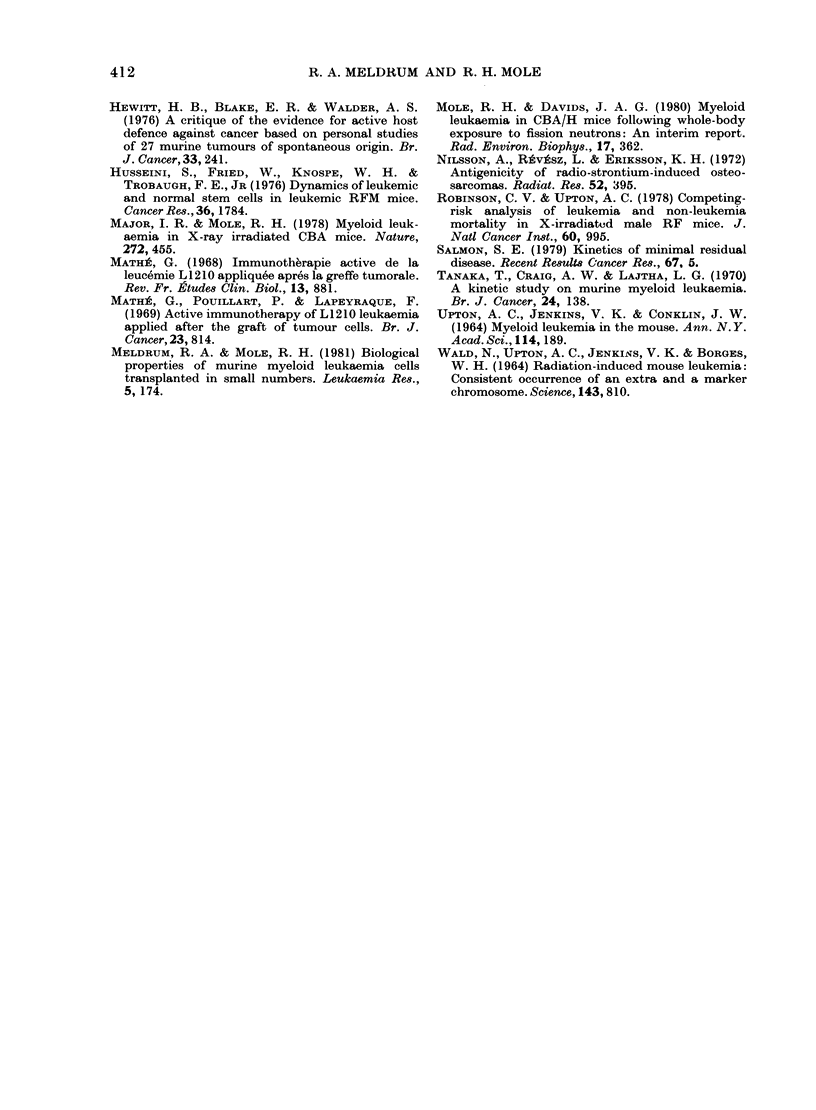# Radiation-induced myeloid leukaemia in CBA/H mice: a non-immunogenic malignant disease in syngeneic mice.

**DOI:** 10.1038/bjc.1982.68

**Published:** 1982-03

**Authors:** R. A. Meldrum, R. H. Mole

## Abstract

In vivo growth characteristics of myeloid leukaemia induced by whole-body irradiation of CBA/H male mice were examined in the strain of origin by procedures expected to enhance or depress immunological responses. Syngeneic growth in vivo (survival time and frequency of takes) was not modified by attempted active immunization with radiation-inactivated cells or by sublethal whole-body irradiation of recipients before inoculation of small numbers of clonogenic cells. Since the growth stimuli involved in in vivo repair of severely damaged normal haemopoietic tissue also did not modify the growth of the radiation-induced leukaemia cells in syngeneic passage, their growth in vivo in the irradiated primary hosts can be regarded as autonomous by the stage at which leukaemia was diagnosed. Challenge inocula in the "immunization" experiments were 1-9 clonogenic cells from 4 different passaged lines and in the whole-body radiation experiments, 1-10(3) clonogenic cells derived from 11 different primary hosts and 4 different passaged lines.


Br. J. Cancer (1982) 45, 403

### RADIATION-INDUCED MYELOID LEUKAEMIA IN CBA/H MICE: A NON-IMMUNOGENIC MALIGNANT DISEASE IN SYNGENEIC MICE

R. A. MELDRUM AND R. H. MOLE

*Visiting Workers, Medical Research Council, Radiobiology Unit, Harwell, Didcot, Oxon OX11 0RD*

Received 3 March 1981 Accepted 9 November 1981

**Summary.—*In vivo* growth characteristics of myeloid leukaemia induced by whole-body irradiation of CBA/H male mice were examined in the strain of origin by procedures expected to enhance or depress immunological responses. Syngeneic growth *in vivo* (survival time and frequency of takes) was not modified by attempted active immunization with radiation-inactivated cells or by sublethal whole-body irradiation of recipients before inoculation of small numbers of clonogenic cells. Since the growth stimuli involved in *in vivo* repair of severely damaged normal haemopoietic tissue also did not modify the growth of the radiation-induced leukaemia cells in syngeneic passage, their growth *in vivo* in the irradiated primary hosts can be regarded as autonomous by the stage at which leukaemia was diagnosed. Challenge inocula in the "immunization" experiments were 1–9 clonogenic cells from 4 different passaged lines and in the whole-body radiation experiments, 1–$10^3$ clonogenic cells derived from 11 different primary hosts and 4 different passaged lines.**

FOR MORE THAN A DECADE a commonly held hypothesis underlying the treatment of malignant disease has been that once the number of malignant cells in the host has been sufficiently reduced by active treatment (surgery, ionizing radiation, chemical agents) the few remaining viable cells can and will all be killed by the body's natural defences, especially its immunological responses (Denoix & Mathé, 1979). The prime experimental evidence came from studies of L1210 leukaemia, a long-maintained cell line which originated in methylcholanthrene-treated DBA/2 mice more than 30 years ago, and was transplanted into a compatible hybrid (C57-BL/6 × DBA/2)$F_1$ (Mathé, 1968; Mathé *et al.*, 1969). However, the experimental end-point was primarily prolongation of survival, not *disappearance* of the leukaemia, and the demonstration of an immunological response to L1210 in $F_1$ hybrids between the strain of origin and other mouse strains (Glynn *et al.*, 1963) may suggest that the experimental model was not wholly satisfactory.

Since myeloid leukaemia can be regularly induced in CBA/H male mice by single brief exposures to X-rays, $\gamma$-rays or fission neutrons over a wide range of doses (Major & Mole, 1978; Mole & Davids, 1980, and unpublished) an alternative model is available for examining questions about the survival and behaviour of small numbers of malignant cells *in vivo*. Attempted active "immunization" and damage to the immune system have been shown to have no effect, as already noted (Meldrum & Mole, 1981).

### METHODS

CBA/Ca mice have been maintained in this laboratory by brother–sister mating for over 30 years and will be referred to subsequently as CBA/H. The diagnosis of myeloid leukaemia was by blood count and film examination, confirmed by subsequent histological preparation of tissues. When grafting leukaemia cells, cell smears were made from another part of the spleen which provided the cell suspension.

Cell suspensions from spleens of leukaemic mice were made in Eagle's solution containing 2–3% serum from normal male CBA/H mice. After slicing with scissors or a knife blade and teasing, a single-cell suspension was easily prepared by syringing the suspension through smaller and smaller needles and then allowing it to stand for a few minutes to allow clumps of cells to settle. Since it is characteristic of myeloid leukaemia in CBA/H mice (unlike lymphoid leukaemia) that single cells in suspension continue to aggregate with the passage of time (sometimes quite rapidly), each stock cell suspension was repeatedly taken up into a syringe and expelled through a 25G needle (as used for the i.v. injections) just before diluting. Each suspension was similarly treated immediately before it was injected into mice. For quantitative experiments, 0·2 ml of 10-fold serial dilutions were injected i.v. each into 5 mice. As a check, the cell concentration in the dilution intended to contain $5 \times 10^5$ cells/ml was determined in a haemacytometer.

Maintenance of multiply passaged lines was usually by i.p. inoculation. If ascitic fluid began to be evident a single i.v. passage usually restored the *status quo*. All recipient mice were male CBA/H mice 90–110 days old, except in the experiments involving active "immunization".

Immunization was attempted by i.v. injection of $10^7$ freshly prepared spleen cells of a multi-passaged myeloid leukaemia line which 30–60 min previously had received 30 or 50 Gy 250 kVp X-rays at 8·4 Gy/min in a plastic container. Three such injections from succeeding but non-consecutive passages of a given line were made into male CBA/H mice, beginning at 60 days of age and then at intervals of 3–6 weeks. Challenge with small numbers of viable leukaemia cells was delayed until 6–10 weeks after the third immunizing injection, to avoid possible confusion by leukaemia originating from cells in the immunizing injections which had escaped inactivation by irradiation. At the outset, before immunization was attempted, pairs of male mice from 5 litters were selected at random, one of each pair to be immunized, the other to be an unimmunized control. They were challenged with the same cell inoculum. Because of the time required for the immunizing procedure, challenge was at 120–160 days of age.

Whole-body irradiation of mice 24 h before inoculation with living leukaemia cells was with 250 kVp X-rays at ~0·5 Gy/min.

The viscera of every dead or dying mouse were examined macroscopically to verify that leukaemia was grossly evident. The spleen was enlarged 5–20-fold, uniformly and without circumscribed foci, usually pink or grey-pink or with brown-green flecks. The liver was uniformly enlarged, but macroscopically not obviously infiltrated. Lymph nodes, peripheral and central, and thymus were not enlarged, except that occasionally cervical nodes were 2–3-fold normal in size, and sometimes pale green. The depth of colour of the sternal marrow and the degree of anaemia were variable. In the experiments reported, there was never any doubt about the cause of deaths within 150 days. Later deaths sometimes needed histological study, particularly when lymphoid leukaemia needed to be excluded. The macroscopic changes were evident but much less conspicuous in deaths following inoculation of cells from multi-passaged lines.

Mice surviving 300 days after challenge or at 200 days after the most delayed positive take, whichever was the later, were killed and examined to confirm the absence of leukaemia.

### RESULTS

### *Grafting of myeloid leukaemia cells*

Myeloid leukaemia has not been observed in over 500 unirradiated controls and, apart from grafted cases, has occurred only in suitably irradiated mice, the primary hosts. One million or more myeloid leukaemia cells taken from the spleen of a freshly killed primary host and inoculated i.v. have not failed to take in 30 attempts. Even when the spleen cells were obtained a number of hours after natural death, the probability of take seemed to be high. Serial dilution of a spleen-cell suspension commonly, but not invariably, gave a linear relationship between survival time and log (number of cells injected i.v.) (Figure). This is what would be predicted from the simplest possible pair of assumptions, that death occurs when the number of leukaemia cells in the body reaches a given value, independently of the number injected, and that after i.v. injection the leukaemia cells

FIGURE.—Survival time and number of i.v. injected spleen cells in specific syngeneic passages in CBA/H male mice of X-ray-induced myloid leukaemia, line XIX. Each point is the average for 5 mice, except where a square bracket indicates a take of < 100%, the subscript number then giving the number of takes in the 5 mice injected (or 10 in the case of the 10 cell injections of P31—see Table I). The vertical bars indicate ± s.e., but for most points s.e. is invisibly small. The cells for P1 (○) came from the spleen of the primary host XIX (receiving 2 doses each of 1·2 Gy of X-rays at 507 and 451 days before the day of passage). $10^7$ P1 cells were also injected i.p. to give P2, from which spleen cells were injected i.p. in larger numbers to give the animal the spleen cells of which were used in experiment P3 (+). This procedure was repeated for experiments P5 (●) and P7 (△), using $10^6$ cells for the first i.p. injection, after which the line has been maintained by i.p. passage of spleen cells for more than 50 generations. Experiments with P1, P7 and P31 are referred to in Tables II, III and I, respectively.

grow *in vivo* exponentially and at a constant rate. The inverse of the slope of the linear relationship is a measure of the mean doubling time of the clonogenic cells involved.

The observed change in frequency of takes with decreasing number of injected cells has been according to Poissonian expectations. It is straightforward, therefore, to calculate from the observations on serial dilutions the number of cells which would give 63% takes; *i.e.*, would fail to give takes in 37% of cases. This corresponds to a Poisson mean of one clonogenic leukaemia cell per injection volume. The inverse of the total number of cells in such a volume is the fraction of leukaemia cells in the suspension which is truly clonogenic, in the sense that leukaemia becomes manifest in a recipient. This effective or clonogenic fraction lay in the range $10^{-2}$–$10^{-4}$ in 14 primary cases of radiation-induced myeloid leukaemia in CBA male mice. The derivation of the clonogenic fraction does not depend on any assumption that all the clonogenic cells have the same growth properties.

When the take frequency was less than or just about 100% the survival times of mice were normally similar, and not dependent on cell number injected, as they are when larger numbers are injected. This is to be expected; when injection volumes contain a Poisson mean of 1 cell or less, mice with positive takes would, with few exceptions, actually have received just one truly clonogenic cell. This expectation about survival times is based on the assumption that all the clonogenic cells in a given spleen suspension have the same growth properties. However, serial passages of minimum numbers of effective cells (*i.e.*, giving < 100% takes) show quite often that different clonogenic cells in a given suspension of spleen cells do not have the same growth properties. This phenomenon is evident in some subsequently described results involving delayed takes (*cf.* Figure and Table III, footnotes § and ||).

When serial inoculations were made, using as the source of the cells on each occasion the spleen of an animal which had received a large number of leukaemia cells, there was a progressive change in growth properties during the first passages (Figure). The survival time for a given number of injected cells decreased, and so did the minimum number of cells required for a take; *i.e.*, the clonogenic fraction increased. Such a progressive change in cellular properties was not seen regularly when spleen cells were cloned *in vivo* by successive inoculation of the minimum effective number of cells, but experiments of this kind are not described here.

TABLE II.—*Influence of whole-body X-irradiation on survival time of syngeneic recipients receiving i.v. inoculations of moderate numbers of cells from the spleen of a CBA/H male mouse which had developed myeloid leukaemia after whole-body irradiation.**

| Myeloid leukaemia serial number | Mean no. of cells injected | Effective cells in inoculum (mean)† | Mean survival (days) | |
|---|---|---|---|---|
| | | | Recipients not X-rayed | Recipients X-rayed‡ |
| XXI | $10^5$ | 1300 | 63 | 65 |
| N1 | ? | ? | 60 | 55 |
| N2 | ? | ? | 58 | 59 |
| XXII | $2 \cdot 5 \times 10^4$ | 50 | 67 | 63 |
| XXVI | $10^4$ | 50 | 59 | 61 |
| XIX | $10^5$ | 30 | $71 \pm 4$ | $59 \pm 2$§ |
| XV | $10^5$ | 30 | 51 | 54 |

* Take frequency always 100%.

† Determined from a calibration curve of frequency of takes in groups of syngeneic male CBA/H mice given 10-fold dilutions of spleen cells. The 95% confidence limits were usually 2–3 × above and below the mean value.

‡ Combined observations from mice receiving 2, 4 or 6 Gy X-rays the day before inoculation, except for XV where the doses were 1·5, 3, 4·5 or 6 Gy.

§ The only statistically established difference between X-rayed and unirradiated recipients ($P < 0{\cdot}01$).

*Attempted immunization by living cells incapable of clonal division*

Mice "immunized" as described with a given serially passaged line of myeloid leukaemia cells were challenged by i.v. injection of 10 unirradiated spleen cells from a further passage of the same line. Their non-immunized brothers each received 10 cells from the same suspension on the same occasion. Three further groups of 5 mice aged 90–110 days were given 1, 10 and 100 cells respectively from the same cell suspension as a calibration set. Table I shows that attempted immunization had no influence on the frequency of takes or on the survival time of mice developing leukaemia, with any of the 4 different lines of myeloid leukaemia tested.

The effective number of truly clonogenic cells in an inoculum of 10 cells was calculated from the frequency of leukaemia takes in the non-immunized mice, and varied from 0·3 to 9 (Table I).

The dose of 30 Gy to 3 successive "immunizing" cell suspensions was insufficient to kill every clonogenic cell of 3 of the 4 leukaemia lines. Thus each of the 4 lines was examined using 50 Gy, and only one (line XVI) with 30 Gy also.

*Whole-body irradiation of recipients*

Five male CBA/H mice which had received 0, 2, 4 or 6 Gy whole-body X-irradiation the day before were given the same i.v. injection of unirradiated spleen cells from leukaemic mice. Cells from 11 primary hosts and from 3 multiply passaged lines were used.

Table II gives the survival times of unirradiated and irradiated mice when the inoculum from a primary host was more than sufficient to kill every recipient with myeloid leukaemia. In 6/7 cases it was clear that prior irradiation of the recipient mice had no influence on survival time. With cells from primary host XIX, the mean survival time was slightly but significantly shorter in the irradiated mice.

Table III gives the results of experiments where the frequency of takes was < 100%. The leukaemia cells came from 4 primary hosts (different from those in Table II) and from 3 passaged lines (2 derived from primary hosts shown in Table II). There was no systematic difference in take frequency between irradiated and unirradiated recipients. There was no systematic difference in survival time in 6/7 experiments. In the

‡ Combined observations from mice receiving 2, 4 or 6 Gy X-rays the day before inoculation.

‖ Mean of 2 takes (226 and 304 days).

¶ The only statistically established difference between X-rayed and unirradiated recipients ($P < 0{\cdot}01$).

7th, with passage 7 of line XIX, the difference in mean survival time between irradiated and unirradiated recipients was suggestive ($P < 0{\cdot}01$). However, survival time was slightly longer for irradiated recipients, the opposite of what was observed with a larger effective inoculum of leukaemia cells from primary host XIX (Table II).

In 2 of the 4 experiments with minimal numbers of clonogenic cells from primary hosts (Table III), the survival time results were straightforward (XXVII and XXVIII) but in the other 2, mean survival time may provide an inadequate summary. Cells from host XXXI gave an exceptionally wide range of survival times for mice developing leukaemia, whether they had been irradiated or not (Table III). For XXX cells there were only 3 leukaemia takes for assessment. The survival time of the unirradiated recipient at 90 days was much shorter than the 226 and 304 days of the 2 takes in irradiated recipients (one after 4 Gy, one after 6 Gy). Overall it seems clear, however, for both primary and passaged myeloid leukaemia cells, that whole-body irradiation did not systematically augment or accelerate the growth of i.v. injected syngeneic cells, even when minimum numbers of clonogenic cells were given.

One methodological problem in interpreting delayed takes in recipients irradiated before inoculation is that whole-body irradiation is itself leukaemogenic. Amongst over 100 cases of leukaemia so caused by single brief exposures to X-rays in the range 1·5–6 Gy, 11 were found before 305 days in 759 mice and none before 226 days. Therefore 2 cases in 15 irradiated recipients of XXX cells at 226 and 304 days are very unlikely to have been caused by the irradiation. The definite myeloid leukaemias of arguable origin in mice receiving XXXI leukaemia cells were at 383 days after 2 Gy and 460 days after 6 Gy, the next most delayed being at 202 days after 2 Gy and 195 days in an unirradiated recipient. This distribution in time, taken at its face value, could well represent an unusually slowly developing class of leukaemia cells. However, if the 2 most delayed cases are discounted as possibly induced by the irradiation of the recipients (not by the cells injected), the degree of change in the results in X-rayed recipients has no bearing on the overall interpretation. Frequency of takes then decreased from 27% to 13% in X-rayed recipients receiving 0·3 effective cells, and mean survival time decreased from 136 to $101 \pm 10$ days, compared with $113 \pm 16$ days in unirradiated mice (Table III).

### DISCUSSION

There are many reports of occasional cases of myeloid leukaemia in groups of laboratory animals treated by a variety of agents, and presumably induced by them but, up to the present, systematic and reproducible induction has been only by ionizing radiation. The first report (concerning irradiated RF mice), was over 40 years ago, and subsequently extensive observations were reported by Upton and his colleagues in the 1950s and 1960s. The disease closely resembled chronic granulocytic leukaemia in man (Upton *et al.*, 1964). The RF mice were not inbred (Robinson & Upton, 1978) and grafting of myeloid leukaemia cells within the strain, even using very large numbers, was not always successful, even after 23–27 passages (Wald *et al.*, 1964). In several respects, this contrasts with our observations on highly inbred CBA/H mice, in which the clinical course and cellular characteristics resemble acute myeloid leukaemia, there is a very low level of the natural disease in unirradiated controls, and syngeneic grafting is straightforward.

It is never possible to provide a conclusive experimental demonstration of the absence of a phenomenon. The evidence presented is the failure to modify the *in vivo* growth characteristics of radiation-induced myeloid leukaemia cells by procedures which should enhance or depress immune responses. Enhancement was attempted by inoculation of large numbers of cells irradiated to prevent their division but not so as to produce immediate and drastic necrosis. Depression of immune responses was by whole-body irradiation at about $\frac{1}{4}$, $\frac{1}{2}$ or $\frac{3}{4}$ $LD_{50}$. The testing procedures were designed to maximize the chance of demonstrating any natural or acquired tumour resistance, by using the smallest possible number of inoculated clonogenic cells, and were all done in a syngeneic situation, to minimize the chances of false positive results. It seems fair to conclude that if the myeloid leukaemia cells are immunogenic *in vivo*, they are very weakly so.

The failure of prior sublethal whole-body irradiation to modify the growth characteristics of the myeloid leukaemia cells *in vivo* must also mean that these cells did not respond to the growth stimuli responsible for the rapid repair of radiation-damaged normal haemopoietic tissue which is so characteristic a sequel of sublethal whole-body exposure. Comparison of irradiated and unirradiated recipients can be regarded as a test for autonomy of growth of the inoculated cells. By such a test the myeloid leukaemia cells used (*i.e.*, within a few days of diagnosis in the primary host) seem truly autonomous. It remains an open question whether this autonomy develops progressively during the "latent interval" between irradiation and diagnosis or is one aspect of the initial transformation of a postulated haemopoietic stem cell into a leukaemia "mother cell".

An apparently variable capacity for growth in a syngeneic host of individual clonogenic cells from the spleen of a primary host can be demonstrated, at least sometimes, when the mean number of effective clonogenic cells in an injection volume $\leq 1$ (*cf.* Table III). There could be a variety of causes, environmental (*e.g.* the chance of a single cell arriving in one micro-environment rather than another in the injected animal) or intrinsic (*e.g.* differences between the genome of different cells which can be revealed only when competition between them can be avoided). The experiments reported here clearly throw little light on such questions.

A "natural immune resistance to neoplastic tissue elements" has been demonstrated in many virally and chemically induced malignancies and in some UV-induced, but remarkably little has been published dealing with tumours induced by ionizing radiation. A large systematic experiment with radiostrontium-induced osteosarcomas, using transplantation into syngeneic recipients previously injected with large numbers of heavily irradiated tumour cells and/or given whole-body irradiation, was interpreted as indicating specific transplantation antigens in the osteosarcomas (Nilsson *et al.*, 1972) but this interpretation can be questioned. Differences were not evident over the whole range of cell inocula, and the basis for the analysis was a quite unusual postulate for the relationship between number of tumour cells inoculated and the take frequency, based not at all on biological considerations but simply on a statistical need for linearity; *viz.*, an arc-sine transformation of the square root of take frequency against log (cell number).

The evidence provided here about radiation-induced myeloid leukaemia is more simply interpretable, and the observation that a regularly reproducible radiation-induced malignancy seems not to be immunogenic may therefore be of interest. Clearly more examples are required for confirmation, not only for myeloid leukaemia but also for other radiation-induced tumours. Even so, as they stand, the findings are in close agreement with the failure to demonstrate a natural defence mechanism against spontaneous tumours (Hewitt *et al.*, 1976) and may be regarded as providing supplementary support for these workers' conclusion that ". . . practically all the animal data presented in support of a general theory of tumour immunogenicity . . . have been obtained from transplanted systems which entail artefactual immunity associated with viral or chemical induction of the tumours or their allogeneic transplantation".

The intrusion of allogeneic phenomena is illustrated in the observations that myeloid leukaemia cells originating in the RFM/UN strain (from Oak Ridge) did not grow in unirradiated RF mice referred to as RFJ (from the Jackson Laboratory) (Husseini *et al.*, 1976) and that they increased exponentially only for a limited period in mice referred to as RF/J (coming from Japan) (Tanaka *et al.*, 1970).

Other reports of the immunogenicity of myeloid leukaemia cells cannot be fully assessed because of inadequate information on the genetic compatibility of cells and the recipients into which they were transplanted. No evidence of resistance to grafts of $10^6$ viable RFM/UN leukaemia cells was found in RFM/UN mice which had been given injections of heat-treated cells of the same origin, but nothing was said about how the originally non-inbred mice had been maintained in the decade before the experiments were done (Adler & Trobaugh, 1978). If immunogenicity was detected for the BNML myeloid leukaemia in Brown Norway rats, it was very weak (Hagenbeek, 1977) and BNML is another line of leukaemia cells which has been maintained by passage for many years in different laboratories and used in animal strains given the same designation but not necessarily with the same genetic constitution.

Myeloid leukaemia in a truly inbred strain of laboratory animal allows less equivocal experimental investigation of possible natural defences against very small numbers of malignant cells ("minimal residual disease") than most experimental models used hitherto. Single-cell suspensions can be prepared very simply from primary hosts, allowing quantitative experiments to be done without the need for potentially damaging procedures for tissue disruption, or for preliminary serial passaging in order to derive cell lines with reproducible growth characteristics but with the concomitant and inescapable selection of only those lines that continue to grow and only those cells that divide fastest. No adjuvant procedure is required to enable small numbers of cells to take. Results are interpretable without reference to the development of a blood supply to a local tumour mass. "Transplantable animal tumours or long passage *in vitro* tumour cell lines may not accurately reflect the proliferative behaviour of the small compartment of tumour stem cells of an individual . . . primary cancer" (Salmon, 1979). Furthermore, the CBA/H myeloid leukaemia cells can be maintained and/or experimented with *in vivo* in a truly syngeneic environment (as if they were continuing to grow in their primary host) avoiding extraneous and possibly misleading immunogenetic influences. It is also easy in practice to use a number of different primaries in any given investigation, so improving the validity of any conclusions that may be drawn.

It is, of course, true that most malignant disease is in the form of a solid tumour with a specific blood supply. However, if it is granted that the only fundamental difference between myeloid leukaemias and solid cancers lies in the need, or lack of it, for a structural component, then more may be learnt about the specific properties of the structural component if an example exists where its influence does not need to be taken into account when investigating the properties of malignant cells as such.

We would like to thank Mr D. G. Papworth for the computations of the Poisson means and their confidence limits and the MRC for financial support.

### REFERENCES

ADLER, S. S. & TROBAUGH, F. E., JR (1978) Splenectomy and immunoprotective treatment in RFM mouse myelogenous leukaemia. *Leukemia Res.*, **2**, 273.

DENOIX, P. & MATHÉ, G. (1979) Introduction. *Recent Results Cancer Res.*, **67**, 1.

GLYNN, J. P., HUMPHREYS, S. R., TRIVERS, G., BIANCO, A. R. & GOLDIN, A. (1963) Studies on immunity to leukemia L1210 in mice. *Cancer Res.*, **23**, 1008.

HAGENBEEK, A. (1977) Introduction of the BN myelocytic leukaemia. *Leukemia Res.*, **1**, 85.

HEWITT, H. B., BLAKE, E. R. & WALDER, A. S. (1976) A critique of the evidence for active host defence against cancer based on personal studies of 27 murine tumours of spontaneous origin. *Br. J. Cancer*, **33**, 241.

HUSSEINI, S., FRIED, W., KNOSPE, W. H. & TROBAUGH, F. E., JR (1976) Dynamics of leukemic and normal stem cells in leukemic RFM mice. *Cancer Res.*, **36**, 1784.

MAJOR, I. R. & MOLE, R. H. (1978) Myeloid leukaemia in X-ray irradiated CBA mice. *Nature*, **272**, 455.

MATHÉ, G. (1968) Immunothèrapie active de la leucémie L1210 appliquée aprés la greffe tumorale. *Rev. Fr. Études Clin. Biol.*, **13**, 881.

MATHÉ, G., POUILLART, P. & LAPEYRAQUE, F. (1969) Active immunotherapy of L1210 leukaemia applied after the graft of tumour cells. *Br. J. Cancer*, **23**, 814.

MELDRUM, R. A. & MOLE, R. H. (1981) Biological properties of murine myeloid leukaemia cells transplanted in small numbers. *Leukaemia Res.*, **5**, 174.

MOLE, R. H. & DAVIDS, J. A. G. (1980) Myeloid leukaemia in CBA/H mice following whole-body exposure to fission neutrons: An interim report. *Rad. Environ. Biophys.*, **17**, 362.

NILSSON, A., RÉVÉSZ, L. & ERIKSSON, K. H. (1972) Antigenicity of radio-strontium-induced osteosarcomas. *Radiat. Res.* **52**, 395.

ROBINSON, C. V. & UPTON, A. C. (1978) Competing-risk analysis of leukemia and non-leukemia mortality in X-irradiated male RF mice. *J. Natl Cancer Inst.*, **60**, 995.

SALMON, S. E. (1979) Kinetics of minimal residual disease. *Recent Results Cancer Res.*, **67**, **5**.

TANAKA, T., CRAIG, A. W. & LAJTHA, L. G. (1970) A kinetic study on murine myeloid leukaemia. *Br. J. Cancer*, **24**, 138.

UPTON, A. C., JENKINS, V. K. & CONKLIN, J. W. (1964) Myeloid leukemia in the mouse. *Ann. N.Y. Acad. Sci.*, **114**, 189.

WALD, N., UPTON, A. C., JENKINS, V. K. & BORGES, W. H. (1964) Radiation-induced mouse leukemia: Consistent occurrence of an extra and a marker chromosome. *Science*, **143**, 810.